# Semen Quality in Transgender Individuals Seeking Fertility Preservation

**DOI:** 10.1111/andr.70268

**Published:** 2026-06-24

**Authors:** Maurizio De Rocco Ponce, Eden Troka, Massimiliano Raffo, Luis Miguel Malca Caballero, Alberto Ferlin, Andrea Salonia, Eduard Ruiz Castañé, Alberto Scala, Andrea Garolla, the Gender Incongruence Interdisciplinary Group (GIIG), Andrea Garolla, Anna Aprile, Camillo Barbisan, Anna Belloni Fortina, Valentina Camozzi, Elena Campello, Annamaria Cattelan, Chiara Ceolin, Fabrizio Dal Moro, Giorgio De Conti, Angela Favaro, Alberto Ferlin, Francesco Francini Pesenti, Michela Gatta, Marta Ghisi, Sandro Giannini, Laura Guazzarotti, Massimo Iafrate, Paolo Meneguzzo, Marina Miscioscia, Giulia Musso, Giancarlo Ottaviano, Corrado Marchese Ragona, Carlo Saccardi, Lolita Sasset, Rossana Schiavo, Giuseppe Sergi, Paolo Simioni, Benedetta Tascini, Eleonora Vania, Elisa Varotto, Francesca Venturini, Tommaso Vezzaro, Fabrizio Vianello

**Affiliations:** ^1^ Fundación Puigvert Servicio De Andrología Fundació Puigvert ‐ IRSantPau Barcelona Spain; ^2^ Department of Medicine University of Padua Padua Italy; ^3^ Department of Systems Medicine Unit of Andrology and Reproductive Medicine University Hospital of Padua Padua Italy; ^4^ Regional Reference Center for Gender Incongruence of the Veneto Region University Hospital of Padua Padua Italy; ^5^ Division of Experimental Oncology, Unit of Urology, URI, IRCCS Ospedale San Raffaele Milan Italy; ^6^ University Vita‐Salute San Raffaele Milan Italy

**Keywords:** fertility preservation, gender‐affirming hormone therapy, reproductive endocrinology, transgender health

## Abstract

**Background:**

Transgender individuals assigned male at birth (AMAB) may choose to preserve their fertility prior to starting gender‐affirming hormone therapy (GAHT). However, limited data exist regarding the baseline reproductive and hormonal characteristics of this population before and after GAHT.

**Objectives:**

To characterize semen quality and hormonal profiles in transgender AMAB individuals prior to GAHT and compare findings with cisgender men and with transgender individuals who discontinued GAHT for at least 3 months.

**Materials and Methods:**

This retrospective study included transgender AMAB individuals from two tertiary andrology centers who underwent sperm cryopreservation before GAHT initiation (treatment‐naïve group). Clinical evaluation included anthropometric measures, testicular volume assessment, varicocele detection, and sex hormone measures. Semen parameters were analyzed according to WHO criteria and compared with those of cisgender men and previously treated transgender individuals.

**Results:**

Thirty‐two treatment‐naïve transgender AMAB individuals were included. Hormonal parameters were generally within reference ranges. Only 46.9% of treatment‐naïve individuals met WHO criteria for normozoospermia, compared with 75.5% of cisgender controls. Compared with nine previously treated individuals, estradiol levels were higher and seminal volume lower. A higher frequency of seminal abnormalities was observed, although not statistically significant.

**Conclusions:**

A substantial proportion of treatment‐naïve transgender AMAB individuals exhibited impaired semen parameters prior to GAHT initiation. Prior GAHT exposure showed a trend toward poorer semen quality. These findings support early fertility counseling and preservation in transgender individuals prior to GAHT initiation.

## 1 Introduction

An increasing number of transgender and gender‐diverse people are seeking gender‐affirming hormone therapy (GAHT) and other medical or surgical interventions to align their physical appearance with their gender identity. For transgender individuals assigned male at birth (AMAB), feminizing hormone therapy consists of estradiol in combination with anti‐androgens, (e.g., cyproterone acetate, spironolactone, or GnRH analogs). Estrogen‐based GAHT is known to negatively impact spermatogenesis, potentially leading to irreversible infertility [1, 2, 3]. As a result, international guidelines recommend that fertility preservation options, such as sperm cryopreservation, be discussed prior to GAHT initiation [3, 4, 5]. However, data on baseline semen quality and hormonal profiles in transgender AMAB individuals before starting GAHT remain limited, and the factors influencing these parameters are not yet fully understood [3, 4].

Existing studies suggest that semen quality in transgender individuals prior to GAHT may already be suboptimal compared to cisgender controls [3]. For example, AMAB often exhibit lower sperm concentration, motility, and morphology even before GAHT initiation [3, 6]. Additionally, clinical and behavioral factors, such as the practice of “tucking” or prolonged use of tight underwear, may further compromise semen quality [4]. The extent and clinical correlates of these findings require further clarification, particularly in young and otherwise healthy populations [3].

The impact of previous GAHT exposure on reproductive function recovery remains debated. While some studies report persistent azoospermia after GAHT cessation, emerging evidence suggests that spermatogenesis may resume following therapy discontinuation [2, 3]. For instance, longitudinal data indicate that viable spermatozoa reappear in some patients after suspending GAHT, challenging the notion of irreversible infertility [2]. Histological evaluation of testicular tissue obtained during vaginoplasty reveals a heterogeneous spectrum, ranging from the complete absence of germ cells to complete spermatogenesis in 24% of cases [7]. Nonetheless, semen parameters in those who temporarily discontinue GAHT often remain poorer than in treatment‐naïve patients, highlighting the need for early fertility counseling [3, 4].

In this context, the present study aims to characterize semen parameters and hormonal profiles in transgender AMAB individuals prior to GAHT and to explore their associations with clinical risk factors [3, 6]. Additionally, by comparing these findings with data from transgender AMAB individuals who discontinued GAHT, we examine whether prior hormone exposure is associated with differences in seminal and hormonal parameters [2, 4]. These insights are critical for optimizing reproductive health outcomes in transgender care.

## 2 Aim of the Study

The present study aims to characterize sperm quality and its clinical and hormonal correlates in transgender AMAB individuals by comparing treatment‐naïve patients with cisgender men and with a transgender AMAB individuals who discontinued GAHT for at least 3 months prior to evaluation.

## 3 Materials and Methods

This is a retrospective multicentric observational study involving two andrological centers: the Unit of Andrology and Reproductive Medicine of the University Hospital of Padua (Italy) and the Unit of Andrology of Fundació Puigvert—Universitat Autònoma de Barcelona (Spain).

The study protocol was approved by both local ethics committees (protocol numbers 2591P and C2025/01).

We included transgender AMAB individuals who performed a sperm cryopreservation in order to preserve fertility prior to initiating GAHT (“treatment‐naïve” group). Exclusion criteria consisted of being under 18 years of age or undergoing active GAHT at the time of sperm collection.

A medical history was collected including pubertal history, use of previous hormonal treatments, smoke habit, and alcohol consumption. All the subjects underwent an accurate physical examination with anthropometric measurements: weight, height, body mass index (BMI), waist circumference (WC), testicular volume (assessed via Prader orchidometer), and presence of varicocele. Blood tests included LH, FSH, total testosterone (TT), estradiol (E2), and prolactin (PRL). We classified the semen analysis according to the sixth edition of the WHO Manual for Human Semen Analysis [8] and defined oligozoospermia as total sperm count < 39 × 10^6^. We defined hypogonadism as TT levels below 8 nmol/L, normal TT levels above 12 nmol/L and a gray‐zone between 8 and 12 nmol/L [9].

“The treatment‐naïve” group cohort was compared with a group of transgender AMAB individuals with a history of previous GAHT who had discontinued the therapy for at least 3 months prior to cryopreservation (“previously treated” group). Seminal parameters were additionally compared with those of a control group of healthy young cisgender men recruited at a blood donation center in Padua (Italy).

### 3.1 Statistical Analysis

The Kolmogorov–Smirnov test was used to assess normal distribution. Continuous variables are presented as median and interquartile range (IQR), and comparison between subgroup was performed with the Wilcoxon–Mann–Whitney test. Categorical variables were expressed as frequencies and percentages and were compared between groups using Pearson's chi squared test. The relationship between continuous variables were evaluated by Spearman's correlation coefficient (ρ). A two‐sided *p* value < 0.05 was considered statistically significant. Statistical analysis was performed using SPSS statistics software for Mac (Version 23; SPSS Inc., Chicago, IL, USA).

## 4 Results

Thirty‐two treatment‐naïve AMAB individuals were included with a mean age of 23 years (IQR 19.2–31.7) and a median BMI of 21.9 kg/m^2^ (IQR 20.1–26.0). Four patients (12.5%) were active smokers, while 7 (21.8%) admitted some kind of alcohol overuse. Finally, 3 patients (9.4%) presented clinical varicocele.

Globally, the semen analysis showed normal seminal volume (2.5 mL, IQR 2.0‐4.0), normal sperm concentration and total count (respectively: 33.2 × 10^6^/mL, IQR 8.1–62.7, and 59.4 × 10^6^, IQR 20.0–129.0), progressive motility in the normal range (43%, IQR 31–58), and normal sperm morphology (6%, IQR 3–8). In detail, 15 patients presented normozoospermia (46.9%), 13 individuals presented some degree of oligozoospermia (of which 5 had isolated oligozoospermia, 3 oligo‐asthenozoospermia, 1 oligo‐teratozoospermia, and 4 oligo‐astheno‐teratozoospermia), 2 patients presented isolated asthenozoospermia, and 2 presented isolated teratozoospermia

Moreover, seminal parameters clustered as follows: seminal volume positively correlated with days of abstinence (ρ = 0.412, *p* = 0.024); sperm concentration and total sperm count showed a positive correlation with progressive motility (ρ = 0.600, *p* < 0.001, and ρ = 0.590, *p* < 0.001, respectively) and morphology (ρ = 0.671, *p* < 0.001 and ρ = 0.655, *p* < 0.001, respectively); progressive motility was positively correlated with normal morphology (ρ = 0.555, *p* = 0.003) and vitality (ρ = 0.497, *p* = 0.042). Correlations are illustrated in the Supporting Information.

With regard to the hormonal status, no patient presented with hypogonadism. The median TT was 20.4 nmol/L (IQR 10.2–25.9) with normal gonadotropins levels: median LH 4.2 U/l (IQR 3.0–7.2) and median FSH 3.8 U/L (IQR 2.5–4.9). They also presented normal E2 and PRL levels: respectively, 60 pmol/L (IQR 31–110) and 12.8 µg/L (IQR 8.6–18.8).

A negative correlation was found between TT and BMI (ρ = ‐0.532, *p* = 0.028) while and a positive correlation was found between TT and LH (ρ = 0.487, *p* = 0.025) and between TT and E2 (ρ = 0.743, *p* < 0.001). Moreover, FSH showed an inverse correlation with sperm concentration (ρ = ‐0.486, *p* = 0.025), sperm total count (ρ = ‐0.674, *p* < 0.001), and morphology (ρ = ‐0.487, *p* = 0.047).

**FIGURE 1:**
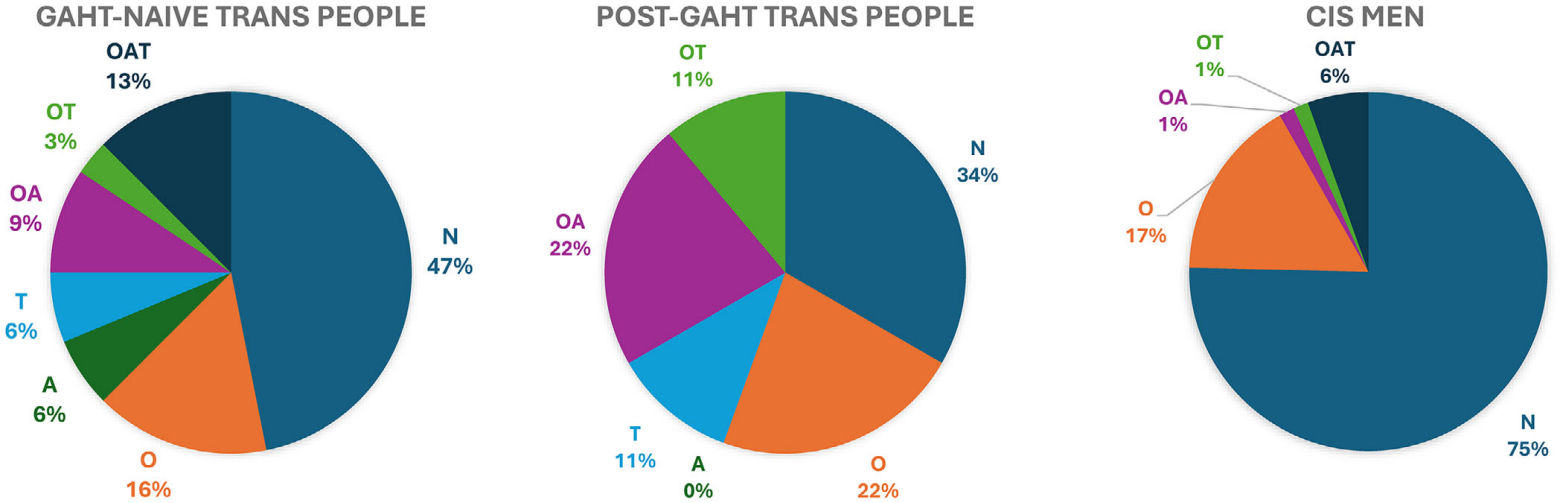
Seminal alterations in treatment‐naïve transgender individuals, previously treated transgender individuals and cisgender men. A, isolated asthenozoospermia; GAHT, gender‐affirming hormone therapy; O, isolated oligozoospermia; OA, oligo‐asthenozoospermia; OAT, oligo‐ astheno‐teratozoospermia; OT, oligo‐teratozoospermia; T, isolated teratozoospermia; N, normozoospermia;

The comparison between treatment‐naïve transgender AMAB individuals and cisgender controls revealed significantly impaired baseline semen parameters in the transgender group. Although treatment‐naïve participants were significantly older than controls (25.2 vs. 19.3 years), BMI did not differ between groups. Treatment‐naïve transgender individuals exhibited significantly lower values across all major spermatogenic parameters, with the exception of seminal volume (Table 1). Overall, seminal abnormalities were more frequent among transgender individuals than among cisgender men (53% vs. 25%, *p* = 0.004). The distribution of seminal alterations across the three groups, namely cisgender men, treatment‐naïve transgender AMAB individuals, and previously treated transgender AMAB individuals, is shown in Figure 1.

**TABLE 1:** Comparison between clinical and seminal parameters in treatment‐naïve transgender individuals and cisgender male controls.

	Trans individuals, treatment naïve (*n* = 32)	Cisgender men (*n* = 74)	*p* value
Age (years)	**25.22 ± 8.23**	**19.33 ± 1.05**	**< 0.0001**
BMI (kg/m^2^)	22.92 ± 5.80	23.14 ± 3.67	0.8858
Seminal volume (mL)	3.09 ± 1.74	2.54 ± 1.41	0.1392
Sperm concentration (M/mL)	**37.60 ± 32.13**	**70.22 ± 48.46**	**0.0004**
Total sperm count (M)	**97.38 ± 122.50**	**172.98 ± 143.71**	**0.0020**
Progressive motility (%)	**41.67 ± 19.61**	**58.78 ± 13.59**	**< 0.0001**
Normal morphology (%)	**5.75 ± 3.76**	**17.40 ± 5.89**	**< 0.0001**

A comparison between naïve and previously treated individuals is summarized in Table 2. The two groups were similar in terms of age, BMI, tobacco and alcohol use, testicular volume and varicocele prevalence. However, previously treated patient presented with lower seminal volume (1.9 mL, IQR 0.6–2.3 vs. 2.5 mL, IQR 2.0–4.0) and higher E2 (150 pmol/L, IQR 120–185, vs. 60 pmol/L, IQR 31–110). Total sperm count and concentration were higher in the treatment‐naïve group, although these differences did not reach statistical significance.

**TABLE 2:** Comparison between clinical and seminal parameters in treatment‐naïve and previously treated transgender AMAB individuals.

	Treatment‐naïve (*N* = 32)	Previously treated (*N* = 9)	*p* value
Age (years)	23.0 (19.2–31.7)	28.0 (24.0–29.0)	0.160
Smoke (*N*, %)	4 (12.5)	4 (44.4)	0.054
Alcohol (*N*, %)	7 (21.8)	2 (22.2)	0.650
Varicocele (*N*, %)	3 (9.4)	0 (0)	0.295
BMI (kg/m^2^)	21.9 (20.1–26.0).	20.5 (19.18–28.7)	0.556
Bi‐testicular volume (mL)	32.0 (25.5–40.0)	28.0 (22.0–35.5)	0.143
Sexual abstinence (days)	4.5 (3.0–5.5)	3.0 (3.0–4.0)	0.070
Seminal volume (mL)	**2.5 (2.0**–**4.0)**	**1.9 (0.6**–**2.3)**	**0.023**
Sperm concentration (M/mL)	33.2 (8.1–62.7)	16.0 (3.6–39.5)	0.404
Total sperm count (M)	59.4 (20.0–129.0)	35.4 (3.4–73.2)	0.152
Progressive motility (%)	43 (31–58)	39.0 (30.0–58.0)	0.771
Sperm morphology (%)	6 (3–8)	6 (1–13)	0.796
Testosterone (nmol/L)	20.4 (10.2–25.9)	25.1 (14.5–32.2)	0.353
LH (U/L)	4.2 (3.0–7.1)	6.2 (4.0–8.0)	0.213
FSH (U/L)	3.8 (2.4–4.9)	3.6 (1.8‐7.6)	0.769
Estradiol (pmol/L)	**60 (31**–**110)**	**150 (120**–**185)**	**0.002**
Prolactin (µg/L)	12.8 (8.6–18.8).	15.7 (14.9–22.4)	0.109

As detailed in Figure 1, the proportion of patients with seminal alterations was higher among previously treated patients, although the difference did not reach statistical significance (67% vs. 47%, *p* = 0.370).

## 5 Discussion

This study is among the few to evaluate semen parameters in a cohort of transgender AMAB individuals before the initiation of GAHT and compare them with individuals previously exposed to GAHT.

On average, treatment‐naïve participants had seminal and hormonal values within normal reference ranges. However, a large proportion of the sample exhibited semen abnormalities: 40.6% had oligozoospermia, 28.1% asthenozoospermia, and 21.9% teratozoospermia. Overall, only 46.9% of the individuals met all WHO criteria for normozoospermia, despite their young age and absence of prior hormonal exposure. This proportion was significantly lower than that observed in our control group of young cisgender men (75%).

Although cisgender controls were, on average, younger than transgender participants, both groups consisted of young adults, suggesting that the age difference is unlikely to be clinically meaningful.

These findings are consistent with previous reports indicating normozoospermia in only 44%–60% of transgender AMAB individuals prior to GAHT initiation [3, 10, 11, 12]. In contrast, data from WHO reference populations and from European cohorts of young cisgender men report more favorable seminal parameters [3, 8, 12, 13, 14, 15].

The causes of these abnormalities remain unclear. Various studies have proposed potential contributing factors, including testicular heat from “tucking” practices or wearing tight clothes [11, 16, 17, 18, 19].

In our study, no significant correlations were found between semen quality and clinical or lifestyle factors such as smoking, alcohol use, BMI, or varicocele. However, higher levels of follicle‐stimulating hormone (FSH) were associated with lower total sperm count and concentration, as well as abnormalities in morphology, suggesting possible subclinical testicular dysfunction. Testosterone levels were generally within normal, even though 5 patients (15.6%) had borderline TT levels between 8 and 12 nmol/L.

Future studies should focus on a more precise evaluation of potential risk factors. In particular, lifestyle habits may play a significant role; research on cardiovascular and bone health has highlighted that transgender individuals often exhibit higher rates of smoking and alcohol consumption, lower levels of physical activity, less healthy dietary habits and weight‐related problems, probably linked to minority stress [20, 21]. Genetic factors may also contribute to these findings. Specifically, a meta‐analysis has found that transgender women on average have more CAG repeats within the polymorphic region of the androgen receptor gene [22]. This genetic variation is linked to decreased androgen sensitivity and may contribute to impaired fertility [23]. However, at the moment, the only risk factor identified is the habit of tucking or wearing tight clothes that may increase testicular temperature [11].

The previously‐treated cohort consisted of a heterogeneous group of transgender patients following different therapeutic approaches. Hormone therapy was primarily based on estradiol, which was prescribed to 8 out of the 9 participants (88.9%). The following antiandrogen therapy was associated: bicalutamide was administered to three individuals (33.3%), cyproterone acetate to two (22.2%), spironolactone to one (11.1%) progesterone in one case (11.1%). One patient received a triple regimen of estradiol, progesterone, and bicalutamide (11.1%). Only one patient (11.1%) received bicalutamide as monotherapy without estradiol.

When comparing treatment‐naïve and previously treated individuals, the latter group showed a higher prevalence of semen abnormalities, although the difference did not reach statistical significance, possibly due to small sample size. Rodriguez‐Wallberg et al. compared semen analysis in AMAB individuals prior to GAHT and after previous treatment, reporting significantly lower sperm concentration, total count, and motility in the treated group [3].

Consistent with previous findings [3], previously treated participants had significantly lower seminal volume. This is likely attributable to the effects of anti‐androgens, such as bicalutamide or cyproterone acetate, which inhibit prostate growth and function [24]. As the prostate contributes substantially to seminal fluid, a reduced prostate volume can lead to lower ejaculate volume [8]. No significant differences in clinical risk factors were observed and hormonal profiles were similar between the two groups, except for estradiol, which was higher in previously treated individuals. This may reflect differences in body composition: estrogen therapy can increase adipose tissue, which in turn promotes peripheral aromatization of testosterone to estradiol [25].

Previous literature has shown that even short‐term GAHT may suppress spermatogenesis, and recovery can be incomplete depending on the duration of therapy and the time elapsed since cessation [26]. In our cohort, the interval between GAHT discontinuation and semen analysis may have been insufficient for full recovery of spermatogenesis in some individuals.

However, the results of the present study should be interpreted with caution due to several limitations. The primary constraint is the small sample size, particularly regarding the group with a history of hormone use. As reported in the literature, only a small fraction of transgender individuals (less than 10%) pursues fertility preservation prior to initiating GAHT [27]. Furthermore, since clinical guidelines prioritize cryopreservation before starting therapy, collecting data from individuals who have already initiated GAHT is even more challenging. For these reasons, no formal power estimation was performed; instead, we aimed to include all available data, a common approach in studies focusing on rare clinical events. This limitation may have reduced the statistical power, potentially preventing the detection of small but clinically relevant differences. Second, the retrospective design introduces potential selection bias and limits the control over confounding variables; specifically, individuals who had suspended therapy had previously used a variety of different GAHT regimens. Finally, the cross‐sectional nature of the analysis precludes definitive conclusions regarding causality or the long‐term recovery of spermatogenesis following GAHT discontinuation. Nonetheless, this study represents one of the few efforts to provide a comprehensive characterization of seminal and hormonal parameters in a well‐defined cohort of transgender AMAB individuals prior to GAHT, while offering a comparison with cisgender controls and a group of previously treated transgender patients. Our results support existing clinical guidelines that recommend offering fertility preservation prior to the initiation of GAHT. Future research should focus on identifying the specific risk factors that influence spermatogenesis and characterizing the long‐term effects of GAHT in the transgender population.

## 6 Conclusion

Our findings reveal a surprisingly high prevalence of semen abnormalities in transgender AMAB individuals prior to GAHT. These results emphasize the importance of individualized fertility assessment and counselling, and they point to the need for further research into the determinants of gonadal health in this population. Compared to prior to GAHT, individuals who have stopped GAHT tend to present a worse semen quality. Larger studies are required to better understand the impact of GAHT on AMAB fertility.

## Author Contributions

Maurizio De Rocco Ponce and Eden Troka were responsible for designing the review protocol, writing the protocol and manuscript, conducting the search, screening potentially eligible studies, extracting and analyzing data, interpreting results, updating reference lists and creating tables and figures. Maurizio De Rocco Ponce and Eden Troka were responsible for designing the review protocol and screening potentially eligible studies. They contributed to writing the report, extracting and analyzing data, and interpreting results. Massimiliano Raffo contributed to data extraction; Luis Miguel Malca Caballero, Alberto Ferlin and Eduard Ruiz Castañé provided feedback on the report. Alberto Scala and Andrea Garolla provided feedback on the report and were also responsible for designing the review protocol, writing the protocol, manuscript and data extraction.

## Funding

The authors have nothing to report.

## Conflicts of Interest

The authors declare no conflicts of interest.

## Supporting information




**Figure S1**: Correlation heatmap of seminal parameters. The heatmap shows the correlation coefficients between key seminal variables, with stronger positive correlations indicated in darker red.
