# Supplementary material for: Semen Quality in Transgender Individuals Seeking Fertility Preservation
Source: Andrology. 2026 Jun 24;14(6):1771–6. doi: 10.1111/andr.70268 (PMC13432531; doi:10.1111/andr.70268)

**Semen quality in transgender individuals seeking fertility preservation**

Maurizio De Rocco Ponce^1#^, Eden Troka^2#^, Massimiliano Raffo^3, 4^, Luis Miguel Malca Caballero^1^, Alberto Ferlin^2^, Andrea Salonia^3^, Eduard Ruiz Castañé^1^, Alberto Scala^2*^, Andrea Garolla^2*^ on behalf of the Gender Incongruence Interdisciplinary Group (GIIG)

^1^Fundación Puigvert, Andrology Department, Universitat Autònoma de Barcelona, Instituto de Investigaciones Biomédicas Sant Pau (IIB-Sant Pau), Barcelona, Spain.

^2^Unit of Andrology and Reproductive Medicine, Department of Medicine (DIMED), University of Padua, Padua, Italy.

^3^Division of Experimental Oncology/Unit of Urology; URI; IRCCS Ospedale San Raffaele, Milan, Italy.

**^4^University Vita-Salute San Raffaele,** Milan, Italy.

^#^The first two authors equally contributed to this work.

^*^The last two authors equally contributed to this article as co-last authors

**CORRESPONDING AUTHOR:**

Maurizio De Rocco Ponce, MD, ORCID ID: 0000-0001-6771-4807

Andrology Department, Fundació Puigvert

Instituto de Investigaciones Biomédicas Sant Pau (IIB-Sant Pau)

Universitat Autónoma de Barcelona, 08041 Barcelona

Email: mderocco@fundacio-puigvert.es

**KEYWORDS:** transgender health, fertility preservation, gender-affirming hormone therapy, reproductive endocrinology.

**Supplementary materials.**

**Figure S1.** Correlation heatmap of seminal parameters. The heatmap shows the correlation coefficients between key seminal variables, with stronger positive correlations indicated in darker red.


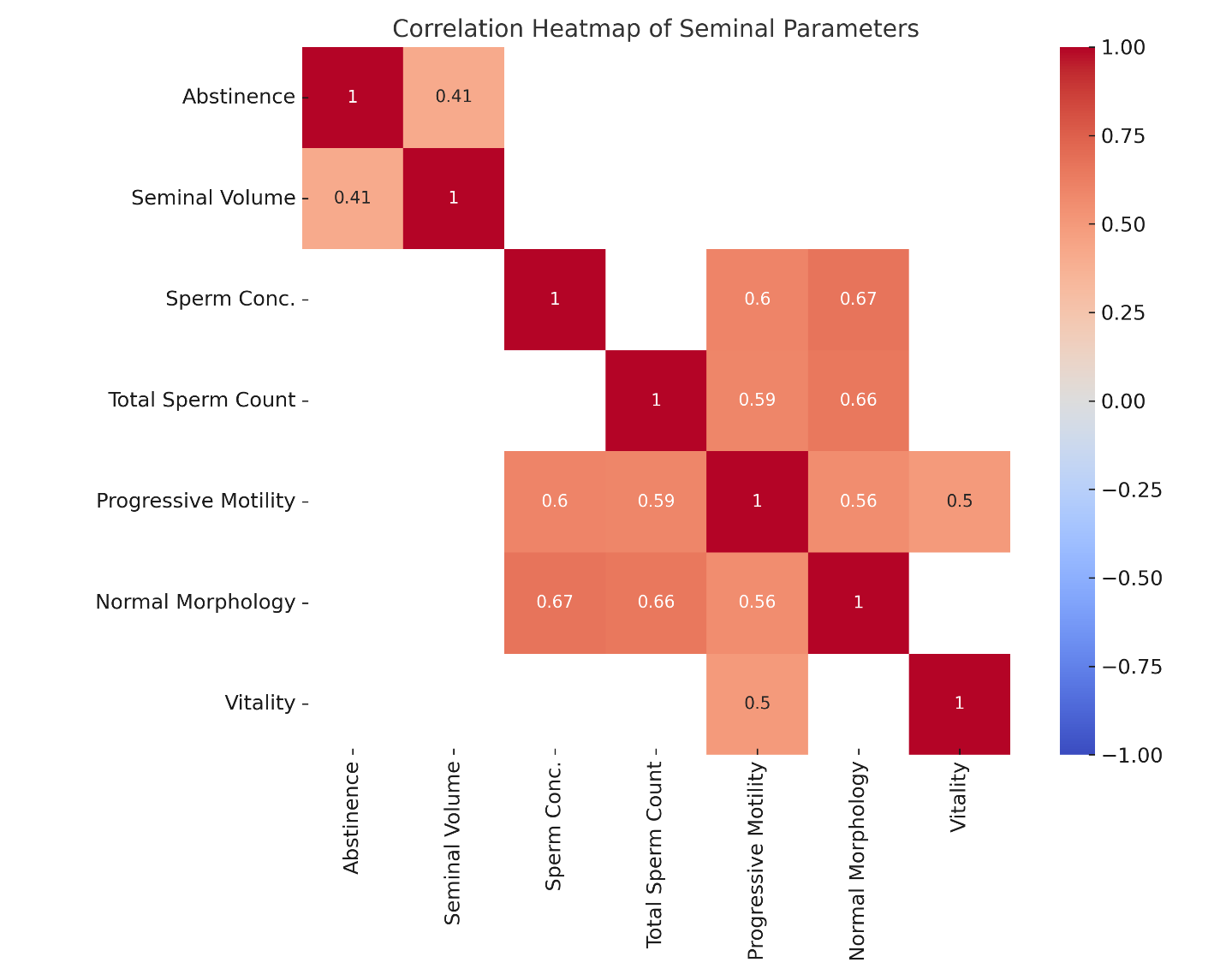

Supplement: Supplementary file 1 — Figure S1: Correlation heatmap of seminal parameters. The heatmap shows the correlation coefficients between key seminal variables, with stronger positive correlations indicated in darker red. [file ANDR-14-1771-s001.docx]
